# Long-term risk of neoplastic events after childhood growth hormone treatment: a population-based cohort study in Sweden

**DOI:** 10.3389/fendo.2024.1360139

**Published:** 2024-03-05

**Authors:** Anders Tidblad, Matteo Bottai, Karin E. Smedby, Kerstin Albertsson-Wikland, Lars Sävendahl

**Affiliations:** ^1^ Department of Women’s and Children’s Health, Karolinska Institutet and Division of Pediatric Endocrinology, Karolinska University Hospital, Solna, Sweden; ^2^ Unit of Biostatistics, Department of Environmental Medicine, Karolinska Institutet, Stockholm, Sweden; ^3^ Division of Clinical Epidemiology (KEP), Department of Medicine Solna, Karolinska Institutet, and Department of Hematology, Theme Cancer, Karolinska University Hospital, Stockholm, Sweden; ^4^ Department of Physiology/Endocrinology, Institute of Neuroscience and Physiology, The Sahlgrenska Academy at the University of Gothenburg, Gothenburg, Sweden

**Keywords:** growth hormone, treatment, cancer, risk, long-term safety, childhood

## Abstract

**Background:**

Increased risk of neoplastic events after recombinant human growth hormone (rhGH) treatment in childhood has been an ongoing concern but long-term safety data are limited.

**Methods:**

A nationwide population-based cohort study in Sweden of patients treated with rhGH during childhood between 1985-2010, due to isolated growth hormone deficiency (GHD), small for gestational age (SGA) and idiopathic short stature (ISS). The comparison group consisted of 15 age-, sex-, and region-matched controls per patient, randomly selected from the general population. Data on neoplastic events and covariates, such as gestational age, birth weight, birth length, socioeconomic status, and height at study start, were collected through linkage with population-based registers. The cohort was followed for neoplastic events until the end of 2020.

**Results:**

53,444 individuals (3,408 patients; 50,036 controls) were followed for up to 35 years, with a median follow-up of 19.8 years and a total of 1,050,977 person-years. Patients showed a moderately increased hazard ratio (HR) for neoplastic events overall compared to controls (HR 1.28, 95% CI: 1.12-1.46), but only significant for males (HR 1.39, 95% CI: 1.17-1.66) and not females (HR 1.15, 95% CI: 0.94-1.41). Longer treatment duration was associated with an increased HR, but no association was found between neoplastic events and mean or cumulative dose. No increased risk of malignant neoplasms was observed for the patients compared to matched controls (HR 0.91 95% CI: 0.66-1.26).

**Conclusion:**

No association was found between rhGH treatment during childhood for GHD, SGA, or ISS and malignant neoplastic events in early to mid-adulthood. A moderate increase in overall neoplastic events was observed due to an increased number of events in male patients.

## Introduction

For over 60 years, growth hormone (GH) has been used clinically, with an increasing number of patients around the world being treated in childhood to achieve increased height, even though they in many cases do not suffer from any secretory hormonal deficiency (1–3). Since its introduction, the use of GH treatment has been accompanied by concerns of potential cancer risks due to its potency as a mitogenic and anti-apoptotic hormone (4). Data from clinical conditions of GH excess (5) or impaired GH signaling (6) has demonstrated a link between GH activity and tumor development and experimental studies using animal models (7) have further elucidated this association. Additionally, epidemiological studies (8) in the general population have shown an association between increased circulating levels of GH’s central mediator, Insulin-like Growth Factor 1 (IGF-1), and certain types of cancer, underscoring the importance of remaining vigilant to this potential risk.

While several studies (9–13) investigating the association between childhood GH treatment and cancer risk have yielded reassuring results, there is still a paucity of studies of long-term risks in this area. The current evidence is based on studies with only a few years of follow-up and lack of proper comparison groups as well as data on potential confounders. With an expanding number of patients starting GH treatment, primarily to improve their stature rather than to replace a secretory hormonal deficiency, the need for valid evidence regarding the long-term implications of this treatment becomes even more pertinent.

The aim of this study was to investigate the risk of neoplasms later in life for patients treated with recombinant human GH (rhGH) in childhood due to isolated growth hormone deficiency (GHD), small for gestational age (SGA), or idiopathic short stature (ISS). To accomplish this objective, we collected outcome data spanning up to 35 years, along with comprehensive information on important covariates including socioeconomic factors, birth characteristics, and height at study start for both the treated patients and a matched comparison group.

## Methods

The overall study design, cohort of rhGH treated patients and the matched comparison group has been described previously in detail (14) and will be briefly summarized.

### Study design, setting and study population

We conducted a nationwide population-based cohort study investigating neoplastic events in Swedish patients treated with rhGH due to GHD, SGA or ISS during childhood between January 1, 1985, and December 31, 2010. The outcome data were prospectively collected from January 1, 1985, to December 31, 2020, and covariates of interest were retrieved through data linkage between Swedish health and population registers. This study was approved by the Regional Ethics Review Board in Stockholm, which waived the need for informed consent for the use of registry data.

Fifteen controls matched for sex, birth year, and geographical region were randomly selected for each patient and linkage of data was achieved using each individual’s unique personal identity numbers (15). As a result, complete data on birth characteristics, health history, vital status, emigration data, educational and income data, as well as socioeconomic data of parents could be linked to each patient and control. Treatment variables such as mean dose, duration of treatment, cumulative dose, and adult treatment, were collected from the GH-SAFETY-database (16) and the Swedish Prescribed Drug Register (17). We also gathered height data for each patient and control from several sources in order to obtain height at study start for each individual.

### Study outcomes

The primary outcome was the detection of the first neoplastic event after the start of the study, defined as the date of first rhGH treatment in the patients or the corresponding date in the matched controls. The secondary outcome was the occurrence of the first malignant neoplastic event. Information on neoplastic events was obtained from the Swedish National Cancer Register (18), the Swedish National Patient Register (19) and from the Cause of Death Register (20), which all have very high national coverage rates. Different categories of neoplastic events were defined according to the ICD codes in the 7^th^-10^th^ revision of the International Classification of Diseases, see **Supplementary Tables S1**, **S2** in **Supplementary Appendix** for the specific ICD codes for each outcome category.

### Statistical analysis

Descriptive statistics were used to summarize baseline characteristics for patients and controls. The incidence rate (IR) of neoplastic events was calculated as the number of new cases observed during the study period divided by the person-time at risk and presented as events per 10,000 person-years with 95% confidence intervals (CIs). IRs were calculated overall and for each stratum of baseline characteristics. Differences in number of events between the patients and controls for each neoplastic category were tested using Fisher’s exact tests.

To assess time to first neoplastic event (overall or only malignant), a Cox proportional hazard model was employed. The assumption of proportional hazards was evaluated using graphical methods and tested using Schoenfeld’s residuals with no significant violation observed. The standard errors were estimated with the cluster sandwich estimator, considering the within-matched-group dependence. The follow-up duration for each participant was calculated from the study start until the date of first neoplastic event or censoring date defined as loss to follow-up (e.g. emigration), death or end of study (December 31, 2020).

The Cox regression analysis was performed with a non-adjusted, a restricted, and a fully adjusted model, to allow a comprehensive evaluation of the relationship between the exposure variable and the time to the outcome event, accounting for potential confounders. The restricted model adjusted for sex, age and height at study start, and the fully adjusted model also included birth length, birth weight, gestational age, parental education, and income. The analysis of time to the first malignant neoplastic event included all subjects, regardless of their prior history of non-malignant neoplasms. All HRs are presented with 95% CIs.

A mixed-effects model was utilized to estimate the height at the start of the study for the control group, taking multiple height measurements for each control subject into account. A detailed description of this model, the sensitivity analyses on a subset of the cohort more similar in height at study start (within 5 cm, **Supplementary Tables S3**, **S4**) or with adult treatment (**Supplementary Table S5**), as well as the other covariates has been reported previously (14). An additional sensitivity analysis with a two-year lag period after end of treatment for all subgroups of patients was also performed (**Supplementary Tables S6**, **S7**). To analyze potential differences in HRs based on follow-up time, a stratified analysis was also conducted, presenting HRs for overall and malignant neoplasms by duration of follow-up (0-9 years, 10-19 years, and ≥20 years) and separated by sex (**Supplementary Table S8**). Kaplan-Meier curves for overall and malignant neoplastic events, separated by sex, are also presented in the **Supplementary Appendix**, **Supplementary Figures S1**, **S2**. A two-sided P value of 0.05 or less was considered statistically significant.

All analyses were performed using Stata statistical software, version 17.0 (StataCorp, TX, USA).

## Results

The study population included 3,408 patients and 50,036 controls (**Table 1**). Mean age at the end of the study was 31.1 years (SD: ± 8.2) with a median follow-up time of 19.8 years (range: 0.0-35.7 years) and a total of 1,050,977 person-years.

**Table 1:** Baseline characteristics of study cohort.

	Patients	Controls	SMD^a^
	(n=3 408)	(n=50 036)	
	n (%)	n (%)	
**Sex**			<0.001
Males	2 305 (67.6)	33 861 (67.7)	
Females	1 103 (32.4)	16 175 (32.3)	
**Gestational age**			0.30
<37w	458 (14.6)	2 596 (5.7)	
37-41w	2 475 (79.0)	39 097 (86.1)	
>42w	199 (6.4)	3 721 (8.2)	
**Birth length SDS** ^b^			0.76
SGA (<-2SDS)	1 075 (35.1)	3 263 (7.3)	
AGA (≥-2 - <+2 SDS)	1 969 (64.3)	39 643 (88.4)	
LGA (≥+2SDS)	18 (0.6)	1 966 (4.4)	
**Birth weight SDS** ^b^			0.55
SGA (<-2SDS)	662 (21.2)	1 884 (4.2)	
AGA (≥-2 - <+2 SDS)	2 441 (78.2)	42 299 (93.5)	
LGA (≥+2SDS)	19 (0.6)	1 062 (2.4)	
**Age at study start**			0.02
0-4 yrs	369 (10.8)	5 517 (11.0)	
5-9 yrs	1 501 (44.0)	21 750 (43.5)	
10-14 yrs	1 433 (42.1)	21 109 (42.2)	
15- yrs	105 (3.1)	1 660 (3.3)	
**Height at study start**			0.93
<100 cm	446 (13.8)	1 195 (2.5)	
100-149 cm	2 698 (83.6)	30 984 (64.9)	
≥150 cm	84 (2.6)	15 600 (32.7)	
**Family income level** ^c^			0.07
**1**	627 (18.5)	9 942 (20.0)	
**2**	638 (18.8)	9 816 (19.7)	
**3**	679 (20.0)	9 963 (20.0)	
**4**	660 (19.4)	10 013 (20.1)	
**5**	792 (23.3)	10 051 (20.2)	
**Parental educational level** ^d^			0.06
**1**	50 (1.5)	929 (1.9)	
**2**	149 (4.4)	2 077 (4.2)	
**3**	864 (25.4)	13 228 (26.5)	
**4**	600 (17.6)	9 274 (18.6)	
**5**	576 (16.9)	8 872 (17.8)	
**6**	1 059 (31.1)	14 229 (28.5)	
**7**	108 (3.2)	1 273 (2.6)	

a SMD, Standardized Mean Differences.

b SDS, Standard Deviation Score; SGA, Small for Gestational Age; AGA, Appropriate for Gestational Age; LGA, Large for Gestational Age.

c Quintiles of total disposable income within the family household at study inclusion, 1=lowest family income quintile and 5=highest family income quintile.

d Highest achievable education level for parents collected from the Swedish Register of Education; 1= primary school <9yrs, 2= primary school 9 yrs, 3= secondary school 0-2 yrs, 4= secondary school >2yrs, 5= higher education <3 yrs, 6= higher education >3 yrs, 7= postgraduate/doctoral studies.

A cumulative count of 9,004 neoplastic diagnoses were registered during follow-up, allowing for multiple diagnoses per individual: 596 in the patient group and 8,408 in the control group (**Supplementary Table S1**). Of these, 1,596 were malignant neoplasms with 84 in the patient group and 1,512 in the control group. The patients had a significantly higher number of neoplasms of uncertain or unknown behavior (104 in the patient group *vs* 1,229 in the control group, p=0.035, **Supplementary Table S1**), with the subcategories of neoplasms in the central nervous system or in unknown sites generating this overall difference (**Supplementary Table S2**). There was no overall difference between the groups concerning benign neoplasms (386 in the patient group *vs* 5,227 in the control group, p=0.106, **Supplementary Table S1**) but the patients had a significantly higher number of benign neoplasms of endocrine glands compared to the controls (13 in the patient group *vs* 51 in the control group, p<0.001, **Supplementary Table S2**). No difference was seen between the groups regarding malignant or benign bone or cartilage neoplasms (**Supplementary Tables S1**, **S2**).

In the analyses of time to first neoplastic event, a total of 7,201 events were recorded (469 in the patient group and 6,552 in the control group). Crude incidence rates (IRs) were similar among patients and controls overall, 69.7 *vs* 66.6 events/10,000 pyrs, as well as for males (55.6 *vs* 51.9 events/10,000 pyrs) and females (103.2 *vs* 101.4 events/10,000 pyrs) separately (**Table 2**). The adjusted hazard ratios (HRs) of overall neoplastic events were slightly increased in both the restricted (HR 1.36, 95% CI: 1.20-1.54) and full model (HR 1.28, 95% CI: 1.12-1.46), predominately in males (**Table 3**). In the sensitivity analysis starting follow-up with a two-year lag-period after end of treatment, no increased risks of overall neoplastic events were noted in the fully adjusted model, except among males in the subgroup of GHD with GH_max_ 5-9 (HR 1.34, 95% CI:1.03-1.74) (**Supplementary Table S6**).

**Table 2:** Number of events, person-years (pyrs) and incidence rates (IRs) for neoplastic events overall (benign and malignant).

	Patients (n=3408)		IR [95% CI]^a^	Controls (n=50036)		IR [95% CI]^a^
	events	pyrs		events	pyrs	
**Total cohort**	469	67 243	69.7 [63.7-76.3]	6 552	983 734	66.6 [65.0-68.2]
Males	263	47 277	55.6 [49.3-62.8]	3 594	692 073	51.9 [50.3-53.7]
Females	206	19 966	103.2 [90.0-118.3]	2 958	291 661	101.4 [97.8-105.1]
Gestational age						
<37w	66	8 366	78.9 [61.2-100.4]	309	50 839	60.8 [54.4-68.0]
37-41w	330	49 491	66.7 [59.9-74.3]	5 249	770 276	68.1 [66.3-70.0]
>42w	38	4 029	94.0 [68.6-129.6]	505	75 914	66.5 [61.0-72.6]
Birth length SDS^b^						
SGA (<-2SDS)	169	21 211	79.7 [68.5-92.6]	439	67 258	65.3 [59.4-71.7]
AGA (≥-2 - <+2 SDS)	250	39 105	63.9 [56.5-72.4]	5 315	780 680	68.1 [66.3-69.9]
LGA (≥+2SDS)	4	317	126.3 [47.4-336.5]	254	39 423	64.4 [57.0-72.9]
Birth weight SDS^b^						
SGA (<-2SDS)	98	13 146	74.5 [61.2-90.9]	232	39 716	58.4 [51.4-66.4]
AGA (≥-2 - <+2 SDS)	331	48 231	68.7 [61.6-76.4]	5 669	833 517	68.0 [66.3-69.8]
LGA (≥+2SDS)	2	333	60.0 [15.0-240.0]	139	20 570	67.6 [57.2-79.8]
Age at study start						
0-4 yrs	49	6 715	73.0 [55.2-96.6]	536	101 285	52.9 [48.6-57.6]
5-9 yrs	197	27 842	70.8 [61.5-81.4]	2 567	406 097	63.2 [60.8-65.7]
10-14 yrs	207	30 205	68.5 [59.8-78.5]	3 183	438 679	72.6 [70.1-75.1]
15- yrs	16	2 482	64.5 [39.5-105.2]	266	37 673	70.6 [62.6-79.6]
Height at study start						
<100 cm	56	8 504	65.8 [50.7-85.6]	144	23 729	60.7 [51.5-71.5]
100-149 cm	388	54 375	71.4 [64.6-78.8]	3 770	593 610	63.5 [61.5-65.6]
≥150 cm	13	1 864	69.7 [40.5-120.1]	2 498	333 396	74.9 [72.0-77.9]
Family income level^c^						
1	75	12 521	59.9 [47.8-75.1]	1 163	191 227	60.8 [57.4-64.4]
2	73	12 824	56.9 [45.3-71.6]	1 192	195 672	60.9 [57.6-64.5]
3	87	13 313	65.3 [53.0-80.6]	1 283	197 562	64.9 [61.5-68.6]
4	97	13 214	73.4 [60.2-89.6]	1 337	199 525	67.0 [63.5-70.7]
5	135	15 211	88.8 [75.0-105.1]	1 563	196 844	79.4 [75.6-83.4]
Parental educational level^d^						
1	6	1 224	49.0 [22.0-109.1]	107	21 603	49.5 [41.0-59.9]
2	22	3 289	66.9 [44.0-101.6]	265	448 630	59.4 [52.6-67.0]
3	109	18 598	58.6 [48.6-70.7]	1 748	277 899	62.9 [60.0-65.9]
4	80	11 175	71.6 [57.5-89.1]	1 102	176 690	62.4 [58.8-66.2]
5	83	11 302	73.4 [59.2-91.1]	1 176	171 771	68.5 [64.7-72.5]
6	147	19 580	75.1 [63.9-88.2]	1 977	265 716	74.4 [71.2-77.8]
7	21	2 039	103.0 [67.1-157.9]	171	23 948	71.4 [61.5-83.0]
Treatment indication subgroups^e^						
SGA (n=672)	100	12 750	78.4 [64.5-95.4]	NA	NA	NA
GHD (n=1 837)	259	37 208	69.6 [61.6-78.6]	NA	NA	NA
ISS (n=899)	110	17 286	63.6 [52.8-76.7]	NA	NA	NA

a Events/10 000 pyrs.

b SDS, Standard Deviation Score; SGA, Small for Gestational Age; AGA, Appropriate for Gestational Age; LGA, Large for Gestational Age.

c Quintiles of total disposable income within the family household at study inclusion, 1=lowest family income quintile and 5=highest family income quintile.

d Highest achievable education level for parents collected from the Swedish Register of Education; 1= primary school <9yrs, 2= primary school 9 yrs, 3= secondary school 0-2 yrs, 4= secondary school >2yrs, 5= higher education <3 yrs, 6= higher education >3 yrs, 7= postgraduate/doctoral studies.

e SGA, Small for Gestational Age; GHD, Growth Hormone Deficiency; ISS, Idiopathic Short Stature. NA, Not Applicable.

**Table 3:** Crude and adjusted hazard ratios for neoplastic events overall (benign and malignant) among patients compared to matched controls.

	N	Crude HR [95% CI]	Adjusted HR [95% CI], restricted model^a^	Adjusted HR [95% CI], full model^b^
**All patients**	3 408	1.05 [0.95-1.15]	1.36 [1.20 -1.54]	1.28 [1.12-1.46]
Male	2 305	1.07 [0.94-1.21]	1.46 [1.24 -1.73]	1.39 [1.17-1.66]
Female	1 103	1.02 [0.88-1.18]	1.24 [1.03-1.50]	1.15 [0.94-1.41]
**SGA** ^c^	672	1.19 [0.98-1.45]	1.55 [1.25-1.93]	1.47 [1.18-1.84]
male	423	1.14 [0.86-1.52]	1.63 [1.20-2.22]	1.54 [1.13-2.10]
female	249	1.17 [0.89-1.55]	1.46 [1.07-1.99]	1.39 [1.01-1.92]
**GHD** ^c^	1 837	1.03 [0.91-1.16]	1.34 [1.16-1.56]	1.26 [1.07-1.48]
male	1 312	1.09 [0.93-1.28]	1.47 [1.21-1.80]	1.42 [1.15-1.75]
female	525	0.98 [0.80-1.19]	1.19 [0.94-1.50]	1.06 [0.82-1.37]
**GHD (GH_max_ 0-4)** ^d^	485	0.96 [0.76-1.21]	1.28 [0.99-1.65]	1.19 [0.91-1.56]
male	349	1.04 [0.76-1.41]	1.46 [1.05-2.02]	1.42 [1.00-2.00]
Female	136	0.90 [0.62-1.31]	1.07 [0.72-1.59]	0.92 [0.60-1.42]
**GHD (GH_max_ 5-9)** ^d^	1 352	1.05 [0.91-1.22]	1.37 [1.16-1.62]	1.29 [1.08-1.54]
male	963	1.12 [0.92-1.35]	1.48 [1.19-1.84]	1.42 [1.13-1.79]
female	389	1.01 [0.81-1.27]	1.24 [0.96-1.61]	1.12 [0.84-1.49]
**ISS** ^c^	899	0.98 [0.81-1.18]	1.26 [1.02-1.55]	1.17 [0.93-1.47]
male	570	0.96 [0.74-1.24]	1.32 [0.99-1.77]	1.21 [0.90-1.64]
female	329	0.98 [0.74-1.28]	1.20 [0.89-1.62]	1.14 [0.81-1.60]
Duration of treatment^e^				
0-2 years	925	0.82 [0.65-1.02]	0.93 [0.72-1.20]	0.89 [0.68-1.17]
3-6 years	1 522	1.01 [0.87-1.16]	1.29 [1.08-1.53]	1.22 [1.01-1.47]
≥7 years	961	0.93 [0.76-1.15]	1.29 [1.03-1.61]	1.23 [0.97-1.55]
*P* trend		0.35	0.03	0.03
Mean GH-dose^e^				
0-29 µg/kg/d	402	1.00 [0.71-1.41]	1.26 [0.88-1.81]	1.20 [0.82-1.74]
30-39 µg/kg/d	2 383	0.89 [0.78-1.01]	1.13 [0.97-1.33]	1.09 [0.92-1.29]
40-49 µg/kg/d	337	0.97 [0.72-1.32]	1.21 [0.87-1.67]	1.13 [0.80-1.59]
≥ 50 µg/kg/d	279	1.20 [0.90-1.60]	1.49 [1.10-2.03]	1.39 [1.01-1.91]
*P* trend		0.19	0.30	0.53
Cumulative dose^e^				
0-1499 mg	1 015	0.82 [0.66-1.02]	0.97 [0.76-1.23]	0.92 [0.72-1.19]
1500-2999 mg	954	1.21 [1.02-1.43]	1.43 [1.17-1.74]	1.37 [1.11-1.69]
3000-4499	902	0.76 [0.62-0.95]	1.06 [0.84-1.34]	0.98 [0.76-1.25]
≥ 4500 mg	381	0.92 [0.69-1.23]	1.39 [1.02-1.88]	1.37 [1.00-1.87]
*P* trend		0.62	0.18	0.22

a Restricted model adjusted only for age at start, height at start and sex (if not stratified for sex).

b Full model adjusted for gestational age, birth length, birth weight, age at start, height at start, parental educational level, family income and sex (if not stratified for sex).

c SGA, Small for Gestational Age; GHD, Growth Hormone Deficiency; ISS, Idiopathic Short Stature.

d GH_max_ = Growth Hormone peak level on either provocation test (mainly Arginine-Insulin Tolerance Test) or during spontaneous 12h or 24h GH secretion profiles (µg/L).

e Analysis with 2-year lag-period after end of treatment to avoid reversed causality (protopathic bias).

In the analyses of detailed treatment exposure, we did not observe any significant trends by different dose categories using mean or cumulative doses (**Table 3**). For duration of treatment, there was a significant trend over duration categories (p_trend_=0.03) with the highest adjusted HRs in the group with the longest (≥7 years) treatment duration but only reaching significance in the restricted model and not in the full model (restricted model: HR 1.29, 95% CI: 1.03-1.61; full model: HR 1.23, 95% CI: 0.97-1.55, **Table 3**).

In the analyses of time to first malignant neoplastic event, a total of 1,381 events were observed (71 in the patients and 1,310 in the controls). Crude IR was lower for the patients compared to the controls (9.9 *vs* 12.6 events/10,000 pyrs) as well as the crude HR (0.78, 95% CI: 0.62-0.99) (**Table 4**). No significant differences were seen in the adjusted analyses with an adjusted HR of 0.93 (95% CI: 0.69-1.25) in the restricted model and 0.91 (95% CI: 0.66-1.26) in the full model. The analysis with a two-year lag-period after end of treatment, confirmed this finding showing no increased HR for malignant neoplastic events among patients compared to controls (**Supplementary Table S7**).

**Table 4:** Incidence rates (IRs) and hazard ratios (HRs) of malignant neoplasms in patients compared to matched controls.

	events	pyrs	IR [95% CI]^a^	Crude HR [95% CI]	Adjusted HR [95% CI], restricted model^b^	Adjusted HR [95% CI], full model^c^
Controls (n=50 036)	1 310	1 038 390	12.6 [11.9-13.3]	1.00 (Ref)	1.00 (Ref)	1.00 (Ref)
Patients (n=3 408)	71	71 662	9.9 [7. 9-12.5]	0.78 [0.62-0.99]	0.93 [0.69-1.25]	0.91 [0.66-1.26]
Male controls (n=33 861)	425	726 023	5.9 [5.3-6.4]	1.00 (Ref)	1.00 (Ref)	1.00 (Ref)
Male patients (n=2 305)	18	50 076	3.6 [2.3-5.7]	0.61 [0.38-0.98]	0.73 [0.42-1.27]	0.73 [0.41-1.32]
Female controls (n=16 175)	885	312 367	28.3 [26.5-30.3]	1.00 (Ref)	1.00 (Ref)	1.00 (Ref)
Female patients (n=1 103)	53	21 586	24.6 [18.8-32.1]	0.86 [0.65-1.14]	1.02 [0.71-1.47]	1.01 [0.68-1.49]

a Events/10 000 pyrs.

b Restricted model adjusted only for age at start, height at start and sex.

c Full model adjusted for gestational age, birth length, birth weight, age at start, height at start, parental educational level, family income and sex.

In the sensitivity analysis, only including patients and controls similar in height at study start, a slight increased HR was detected for neoplastic events overall in the patient group in the restricted model (HR 1.32, 95% CI: 1.01-1.72) but not reaching significance in the full model (HR 1.31, 95% CI: 0.97-1.77) ( **Supplementary Table S4**). Similar non-significant point estimates were seen for malignant neoplastic events but with even wider confidence intervals due to few events (**Supplementary Table S4**). The analysis of patients with adult treatment showed increased HRs for overall neoplastic events but not for malignant neoplastic events (**Supplementary Table S5**). The stratified analyses of HRs for overall and malignant neoplasms by duration of follow-up did not detect any clear differences in risk based on follow-up duration (**Supplementary Table 8**; **Supplementary Figures S1**, **S2**).

## Discussion

This nationwide population-based cohort study of Swedish patients treated with rhGH in childhood due to GHD, ISS or SGA with up to 35 years of follow-up, did not detect an increased risk of malignant neoplastic events. Regarding overall neoplastic events, including benign and unspecific neoplasms, a moderately increased occurrence in the patient group was seen, caused by an increased risk in male patients, and for those with the longest duration of treatment. This increase was caused by a higher frequency of benign and unspecific neoplasms and markedly diminished in the sensitivity analysis introducing a two-year lag-period after end of treatment. The present study thus supports the overall safety of rhGH-treatment to pediatric patients with GHD, ISS or SGA regarding the long-term risk of neoplastic events, and in particular regarding the risk of malignant neoplasms.

The potential cancer risk associated with GH treatment has been an ongoing concern since its introduction due to the role of the GH-IGF-1 signaling pathways in fundamental cellular processes of mitosis, growth and cell survival, along with supporting experimental evidence of its impact on tumor formation (21, 22). In the late 1980s, increased risk of leukemia associated with GH-treatment was reported from Japan (23) but could not be confirmed in later studies showing no increased risk in patients without previous risk factors (24). In 2002, a cohort study of patients previously treated with human pituitary-derived GH reported an increased relative mortality due to colorectal cancer and Hodgkin lymphoma but based on very few cases (25).

Large post-marketing databases have been initiated to detect adverse event signals, including neoplasms, in rhGH-treated patients. Most publications from these cohorts align with our study, showing no increased risk of cancer incidence or mortality in patients without an underlying increased cancer risk (9, 12, 26–30). Although these databases have gathered significant patient-years, the average follow-up is only about four years, which restricts conclusions on long-term risks. Given the typically prolonged latency period for malignancy development, a comprehensive follow-up period is necessary to detect any potential association with prior GH treatment.

Addressing some of the limitations in previous studies, a joint collaboration of eight European countries (SAGhE) was initiated in 2009 (31). In 2012, a publication from the French sub-cohort of GHD, ISS and SGA patients, reported no overall increased cancer-related deaths but an increased site-specific mortality due to bone tumors, based on three fatal cases (32). The final analysis of the whole SAGhE-cohort, similarly reported an increased site-specific mortality for bone tumors, based on the three included French cases, but no overall increased cancer mortality or increased cancer incidence in the GHD, ISS or SGA patients (13). In a subsequent study involving the same French sub-cohort, an additionally elevated incidence of bone tumors was reported (33).

Despite the extended follow-up period of around 17 years in the SAGhE-based publications, methodological challenges persist, preventing the ability to draw firm conclusions regarding the long-term cancer risks. The cohorts are largely heterogenous, lack information on adult treatment, and only crude comparisons with the general population were performed with no information of potential confounding factors or adjusted analyses taking such factors into account. The present study attempts to address several of these issues with a randomly selected and matched comparison group as well as data on important covariates such as birth characteristics, socioeconomic factors and height at study start for both the patients and controls. By controlling for all these factors, amongst other unmeasurable height-associated confounding factors, we aimed, as far as possible, to isolate how rhGH-treatment exposure in childhood might affect future risk of neoplasia.

In the present study, we only observed a moderate rise in the risk of overall neoplastic events and no increased risk of malignant neoplasms or tumors, benign or malignant, in bone or cartilage tissue. In the dose-response analyses, an overall significant trend with longer treatment duration was observed but not for higher mean or cumulative doses. If a higher exposure to rhGH treatment was indeed associated with an increased risk for future neoplastic events, one would expect to observe a similar trend for cumulative dose as well. Our stratified analysis, examining potential increases in risks of neoplasms based on follow-up length, also failed to detect any discernible difference, further reinforcing our overall findings.

In a previous study on GHD patients treated with rhGH as adults, Child et al. reported increased relative risks of primary cancer in those with a childhood onset GHD and those in the lowest age group (<35 years) (34). In our subgroup of adult-treated patients, we could not see an increase in malignant neoplasms, only an increased risk for overall neoplastic events indicating that this finding is underpinned by benign or uncertain neoplasms.

Despite considerable efforts to address numerous methodological challenges associated with investigating this subject, our study also has limitations, the foremost being the absence of untreated controls. However, instead of relying solely on comparisons with the general population, we established a comparison group that closely resembled our patients to isolate the effect of rhGH-treatment exposure. This was achieved by randomly selected age-, sex- and region-matched controls with adjustment of multiple potential confounders such as birth characteristics, socioeconomic factors, and height at study start. Secondly, a risk of detection bias could be present in our study, possibly explaining the increased number of benign tumors in endocrine glands, and we have addressed this by adding sensitivity analyses with a lag-period not only for the dose-response analyses, but also for all subgroup analyses. We could see that this analysis diminished any differences between the groups even further, reinforcing our main finding of similar neoplastic risks between patients and controls. Thirdly, in some of our subgroup analyses we had few events which created some uncertainty regarding our reported point estimates and increases the risk of type II errors. However, most of our analyses exhibit adequate statistical power, supporting the validity of our reported findings. Lastly, even if this is to date the longest follow-up of childhood rhGH-treated patients, the relative youth of our cohort restrict us to infer about risks in later adulthood and further motivates continuous surveillance of these patients into older ages.

In this nationwide population-based cohort study conducted in Sweden, encompassing a follow-up period of up to 35 years for children treated with rhGH due to GHD, ISS or SGA, we did not detect an increased risk of malignant neoplastic events in early to mid-adulthood. Only a moderate increase in overall neoplastic events was observed for a subgroup of patients, reinforcing our overall reassuring results. While continued monitoring of previously treated patients is still necessary, the present study represents the most comprehensive evidence available to date regarding the long-term cancer safety of rhGH treatment for the above-mentioned indications.

## Data availability statement

The datasets presented in this article are not readily available because the collected data for this study, received from the National Board of Health in Sweden and Statistics Sweden, cannot be shared with third parties based on legal agreements in data delivery and in accordance with prevailing laws and regulations governing the management of personal data in Sweden.

## Ethics statement

The studies involving humans were approved by the Regional Ethics Review Board in Stockholm (dnr: 2010/578-31/1, 2011/109-32-1, 2011/305-32, 2014/1775-32 and 2017/515-32). The studies were conducted in accordance with the local legislation and institutional requirements. The ethics committee/institutional review board waived the requirement of written informed consent for participation from the participants or the participants’ legal guardians/next of kin because the study only used registry data.

## Author contributions

AT: Conceptualization, Data curation, Formal analysis, Funding acquisition, Investigation, Methodology, Project administration, Resources, Software, Supervision, Validation, Visualization, Writing – original draft, Writing – review & editing. MB: Data curation, Formal analysis, Methodology, Supervision, Validation, Writing – review & editing. KS: Methodology, Supervision, Writing – review & editing. KA-W: Data curation, Methodology, Supervision, Validation, Writing – review & editing. LS: Data curation, Funding acquisition, Methodology, Project administration, Supervision, Validation, Writing – review & editing.
