# Supplementary material for: Long-term risk of neoplastic events after childhood growth hormone treatment: a population-based cohort study in Sweden
Source: Front Endocrinol (Lausanne). 2024 Mar 5;15:1360139. doi: 10.3389/fendo.2024.1360139 (PMC10948557; doi:10.3389/fendo.2024.1360139)

Supplementary Appendix:

Long-term risk of neoplastic events after childhood growth hormone treatment: a population-based cohort study in Sweden

Table S1. ICD-codes and number of cases for each neoplastic category

Table S2. Subcategorization for benign neoplasms and neoplasms of uncertain or unknown behavior and number or cases within each subcategory

Table S3. Basal characteristics for the patients and controls in the sensitivity analysis on a subset of patients and controls more similar in height (within 5 cm) at study start

Table S4. Number of events, person-years (pyrs), incidence rate ratios (IRRs), crude and adjusted hazard ratios (HRs) for overall and malignant neoplasms in the sensitivity analysis on a subset of patients and controls more similar in height (within 5 cm*)* at study start

Table S5. Incidence rate ratios (IRRs), crude and adjusted hazard ratios (HRs) for overall and malignant neoplasms among individuals prescribed rhGH-treatment after 18 years of age in the Prescribed Drug Register (2005-2020)

Table S6. Crude and adjusted hazard ratios for all neoplastic events with two-year lag period after end of treatment

Table S7: Crude and adjusted hazard ratios for malignant neoplasms with two-year lag period after end of treatment

Table S8: Hazard ratios for overall and malignant neoplasms by duration of follow-up, separated by sex

Figure S1: Kaplan-Meier curves of overall neoplastic events, separated by sex

Figure S2: Kaplan-Meier curves of malignant neoplastic events, separated by sex

Table S1: ICD-codes and number of cases for each neoplastic category

Neoplastic category	ICD7	ICD8	ICD9	ICD10	Patients	Controls	P-value[a]
Malignant neoplasms of lip, oral cavity, and pharynx	140-148	140-149	140-149	C00-C14	0	12	1.000
Malignant neoplasms of digestive organs	150-159	150-159	150-159	C15-C26	2	58	0.591
Malignant neoplasms of respiratory and intrathoracic organs	160-165	160-163	160-165	C30-C39	1	15	1.000
Malignant neoplasms of bone and articular cartilage	196-197	170-171	170-171	C40-C41	2	27	0.708
Melanoma and other malignant neoplasms of skin	190-191	172-173	172-173	C43-C44	10	215	0.274
Malignant neoplasms of mesothelial and soft tissue				C45-C49	1	21	1.000
Malignant neoplasm of breast	170	174	174	C50-C50	1	29	1.000
Malignant neoplasms of female genital organs	171-176	180-184	180-184	C51-C58	39	733	0.138
Malignant neoplasms of male genital organs	177-179	185-187	185-187	C60-C63	5	92	0.835
Malignant neoplasms of urinary tract	180-181	188-189	188-189	C64-C68	0	15	0.620
Malignant neoplasms of eye, brain, and other parts of central nervous system	192-193	190-192	190-192	C69-C72	4	67	1.000
Malignant neoplasms of thyroid and other endocrine glands	194-195	193-194	193-194	C73-C75	6	47	0.150
Malignant neoplasms of ill-defined, secondary, and unspecified sites	198-199	195-199	195-199	C76-C80	5	84	1.000
Malignant neoplasms, stated or presumed to be primary, of lymphoid, hematopoietic, and related tissue	200-207	200-209	200-208	C81-C96	8	97	0.548
Malignant neoplasms of independent (primary) multiple sites				C97-C97	0	0	NA
In situ neoplasms			230-234	D00-D09	22	440	0.180
Benign neoplasms	210-229	210-228	210-229	D10-D36	386	5227	0.106
Neoplasms of uncertain or unknown behavior	230-239	230-239	235-239	D37-D48	104	1229	0.035

[a] Fischer exact test

Table S2: Subcategorization for benign neoplasms and neoplasms of uncertain or unknown behavior and number or cases within each subcategory

Benign neoplasms	ICD7	ICD8	ICD9	ICD10	Patients	Controls	P-value[a]
Benign neoplasm of mouth, pharynx, and major salivary glands	210	210	210	D10-D11	4	110	0.189
Benign neoplasm of colon, rectum, anus, anal canal, and other parts of digestive system	211	211	211	D12-D13	4	51	0.789
Benign neoplasm of middle ear, respiratory system, and other intrathoracic organs	212	212	212	D14- D15	3	23	0.420
Benign neoplasm of bone and articular cartilage	225	213	213	D16	21	222	0.245
Benign lipomatous neoplasm	226	214	214	D17	15	282	0.238
Hemangioma and lymphangioma	228	227	228	D18	8	181	0.186
Benign neoplasm of mesothelial, connective, and other soft tissue	227	215	215	D19-D21	20	288	0.908
Melanocytic nevi and other benign neoplasms of skin	220, 222	216	216	D22-D23	286	3871	1.000
Benign neoplasm of breast	213	217	217	D24	9	129	1.000
Benign neoplasms of uterus	214-215	218-219	218-219	D25-D26	5	123	0.217
Benign neoplasm of ovary and other female genital organs	216-217	220-221	220-221	D27-D28	10	106	0.455
Benign neoplasm of male genital organs	218	222	222	D29	1	25	1.000
Benign neoplasm of urinary organs	219	223	223	D30	1	4	0.300
Benign neoplasm of eye and adnexa		224	224	D31	9	62	0.059
Benign neoplasm of meninges, brain, and other parts of central nervous system	223	225	225	D32-D33	5	45	0.391
Benign neoplasm of endocrine glands	224	226	226-227	D34-D35	13	51	<0.001
Benign neoplasm of other and unspecified sites	229	228	229	D36	3	40	1.000

Table S2: Subcategorization for benign neoplasms and neoplasms of uncertain or unknown behavior and number or cases within each subcategory (continued)

Neoplasms of uncertain or unknown behaviour	ICD7	ICD8	ICD9	ICD10	Patients	Controls	P-value[a]
Neoplasm of uncertain or unknown behavior of oral cavity and digestive organs	230	230	235	D37	10	105	0.715
Neoplasm of uncertain or unknown behavior of middle ear and respiratory and intrathoracic organs	231	231	231	D38	3	27	0.504
Neoplasm of uncertain or unknown behavior of female genital organs	233-235	234-236	236	D39	2	20	0.688
Neoplasm of uncertain or unknown behavior of male genital or urinary organs	236	237	236	D40-41	10	94	0.447
Neoplasm of uncertain or unknown behavior of meninges, brain, and central nervous system	237	238	237F-X	D42-43	9	43	0.016
Neoplasm of uncertain or unknown behavior of endocrine glands			237A-E	D44	5	52	0.799
Neoplasm of uncertain or unknown behavior of other and unspecified sites	232, 238, 239	232, 233, 239	238-239	D45-48	66	905	0.029

[a] Fischer exact test

Table S3: Basal characteristics for the patients and controls in the sensitivity analysis on a subset of patients and controls more similar in height (within 5 cm) at study start

	Patients	Controls	SMD [a]
	(n=837)	(n=1,695)	
**Sex**			0.06
Males, n (%)	583 (69.7)	1,131 (66.7)	
Females, n (%)	254 (30.3)	564 (33.3)	
**Gestational age (week)**			0.21
Mean (SD)	38.5 (3.0)	39.1 (2.2)	
**Birth length (SDS)**			0.30
Mean (SD)	-1.30 (1.4)	-0.90 (1.3)	
**Birth weight (SDS)**			0.24
Mean (SD)	-0.81 (1.3)	-0.52 (1.1)	
**Age at start (years)**			-0.11
Mean (SD)	7.94 (2.99)	7.60 (3.26)	
**Height at start (cm)**			0.15
Mean (SD)	115.6 (17.5)	118.3 (19.7)	
**Family income level**			-0.29
Mean (SD)	3.2 (1.4)	2.8 (1.4)	
**Parental educational level**			-0.26
Mean (SD)	4.7 (1.4)	4.4 (1.4)	

[a] SMD=Standard Mean Differences

Table S4: Number of events, person-years (pyrs), incidence rate ratios (IRRs), crude and adjusted hazard ratios (HRs) for overall and malignant neoplasms in the sensitivity analysis on a subset of patients and controls more similar in height (within 5 cm) at study start

	Events (n)	Pyrs	IRR [95% CI]	Crude HR [95% CI]	*p*-value	Adjusted HR [95% CI], restricted model[a]	*p*-value	Adjusted HR [95% CI], full model[b]	*p*-value
**Overall neoplasms**									
Controls (n=1,695)	196	32826	1.00 (Ref)	1.00 (Ref)		1.00 (Ref)		1.00 (Ref)	
Patients (n=837)	111	16089	1.16 [0.91-1.47]	1.16 [0.91-1.46]	0.226	1.32 [1.01-1.72]	0.045	1.31 [0.97-1.77]	0.073
**Malignant neoplasms**									
Controls (n=1,695)	37	34458	1.00 (Ref)	1.00 (Ref)		1.00 (Ref)		1.00 (Ref)	
Patients (n=837)	18	17087	0.98 [0.53-1.77]	0.98 [0.57-1.68]	0.931	1.38 [0.64-2.98]	0.414	1.91 [0.78-4.70]	0.157

[a] Restricted model adjusted only for age at start, height at start and sex.

[b] Full model adjusted for gestational age, birth length, birth weight, age at start, height at start, parental educational level, family income and sex.

Table S5: Incidence rate ratios (IRRs), crude and adjusted hazard ratios (HRs) for overall and malignant neoplasms among individuals prescribed rhGH-treatment after 18 years of age in the Prescribed Drug Register (2005-2020)

	Events (n)	Pyrs	IRR [95% CI]	Crude HR [95% CI]	*p*-value	Adjusted HR [95% CI], restricted model[b]	*p*-value	Adjusted HR [95% CI], full model[c]	*p*-value
**Overall neoplasms**									
Controls[a] (n=46,654)	6,383	946,581	1.00 (Ref)	1.00 (Ref)		1.00 (Ref)		1.00 (Ref)	
Patients (n=191)	34	3608	1.40 [0.97-1.95]	1.45 [1.03-2.03]	0.034	2.16 [1.52-3.08]	<0.001	2.13 [1.47-3.09]	<0.001
**Malignant neoplasms**									
Controls[a] (n=46,654)	1305	999,830	1.00 (Ref)	1.00 (Ref)		1.00 (Ref)		1.00 (Ref)	
Patients (n=191)	3	3951	0.58 [0.12-1.70]	0.61 [0.20-1.88]	0.386	1.03 [0.33-3.23]	0.956	1.21 [0.39-3.77]	0.738

[a] Only controls with same age range (18-52 years) kept as comparison group to exclude difference of age range between the patients and controls.

[b] Restricted model adjusted only for age at start, height at start and sex.

[c] Full model adjusted for gestational age, birth length, birth weight, age at start, height at start, parental educational level, family income and sex.

Table S6: Crude and adjusted hazard ratios for all neoplastic events with two-year lag period after end of treatment

	N	Crude HR [95% CI]	Adjusted HR [95% CI], restricted model[a]	Adjusted HR [95% CI], full model[b]
**All patients**	3408	0.94 [0.85-1.05]	1.20 [1.04 -1.38]	1.14 [0.98-1.33]
Male	2305	0.91 [0.79-1.06]	1.24 [1.02 -1.51]	1.18 [0.96-1.44]
Female	1103	0.98 [0.84-1.14]	1.14 [0.93-1.40]	1.10 [0.88-1.37]
**SGA**[c]	672	1.02 [0.81-1.28]	1. 26 [0.98-1.61]	1.19 [0.92-1.54]
male	423	0.85 [0.59-1.21]	1.18 [0.80-1.73]	1.12 [0.76-1.65]
female	249	1.11 [0.82-1.50]	1.29 [0.92-1.81]	1.23 [0.86-1.75]
**GHD**[c]	1837	0.92 [0.79-1.06]	1.18 [1.00-1.40]	1.12 [0.93-1.34]
male	1312	0.93 [0.77-1.13]	1.26 [1.00-1.59]	1.19 [0.93-1.51]
female	525	0.94 [0.76-1.17]	1.10 [0.86-1.42]	1.04 [0.79-1.37]
**GHD (GH_{max} 0-4)**[d]	485	0.72 [0.53-0.96]	0.94 [0.69-1.28]	0.87 [0.62-1.21]
male	349	0.62 [0.41-0.95]	0.87 [0.56-1.36]	0.81 [0.51-1.30]
female	136	0.89 [0.59-1.35]	1.03 [0.67-1.58]	0.94 [0.59-1.49]
**GHD (GH_{max} 5-9)**[d]	1352	1.00 [0.85-1.17]	1.28 [1.06-1.54]	1.22 [1.00-1.49]
male	963	1.06 [0.86-1.32]	1.42 [1.11-1.83]	1.34 [1.03-1.74]
female	389	0.96 [0.75-1.24]	1.13 [0.85-1.50]	1.09 [0.80-1.48]
**ISS**[c]	899	0.94 [0.76-1.16]	1.18 [0.94-1.49]	1.15 [0.89-1.47]
male	570	0.91 [0.68-1.23]	1.25 [0.90-1.74]	1.20 [0.85-1.68]
female	329	0.93 [0.70-1.25]	1.12 [0.82-1.54]	1.11 [0.77-1.60]

[a] Restricted model adjusted only for age at start, height at start and sex (if not stratified for sex)

[b] Full model adjusted for gestational age, birth length, birth weight, age at start, height at start, parental educational level, family income and sex (if not stratified for sex)

[c] SGA=Small for Gestational Age, GHD=Growth Hormone Deficiency, ISS=Idiopathic Short Stature.

[d] GH_{max} = Growth Hormone peak level on either provocation test (mainly Arginine-Insulin Tolerance Test) or during spontaneous 12h or 24h GH secretion profiles (µg/L).

Table S7: Crude and adjusted hazard ratios for malignant neoplasms with two-year lag period after end of treatment

	events	pyrs	IR [95% CI][a]	Crude HR [95% CI]	Adjusted HR [95% CI], restricted model[b]	Adjusted HR [95% CI], full model[c]
Controls (n=50036)	1247	679374	18.4 [17.4-19.4]	1.00 (Ref)	1.00 (Ref)	1.00 (Ref)
Patients (n=3408)	64	47104	13.6 [10.6-17.4]	0.74 [0.58-0.95]	0.88 [0.65-1.20]	0.86 [0.62-1.21]
Male controls (n=33861)	372	471290	7.9 [7.1-8.7]	1.00 (Ref)	1.00 (Ref)	1.00 (Ref)
Male patients (n=2305)	15	32643	4.6 [2.8-7.6]	0.58 [0.35-0.97]	0.75 [0.41-1.36]	0.72 [0.38-1.37]
Female controls (n=16175)	875	208085	42.1 [39.4-44.9]	1.00 (Ref)	1.00 (Ref)	1.00 (Ref)
Female patients (n=1103)	49	14461	33.9 [25.6-44.8]	0.80 [0.60-1.07]	0.93 [0.64-1.33]	0.92 [0.62-1.37]

[a] Events/10,000 pyrs

[b] Restricted model adjusted only for age at start, height at start and sex

[c] Full model adjusted for gestational age, birth length, birth weight, age at start, height at start, parental educational level, family income and sex

Table S8: Hazard ratios for overall and malignant neoplasms by duration of follow-up, separated by sex

	HR [95% CI] [a]		
	**Follow-up duration (0-9 yrs)**	**Follow-up duration (10-19 yrs)**	**Follow-up duration (≥20 yrs)**
**Overall neoplastic events**			
All patients	1.15 [0.90-1.47]	1.22 [1.01-1.47]	1.25 [0.96-1.64]
Males	1.26 [0.93-1.71]	1.28 [0.99-1.67]	1.18 [0.84-1.67]
Females	0.97 [0.65-1.47]	1.18 [0.90-1.57]	1.36 [0.88-2.09]
**Malignant neoplastic events**			
All patients	1.38 [0.46-4.11]	0.82 [0.53-1.29]	1.29 [0.79-2.09]
Males	1.05 [0.28-3.94]	0.21 [0.05-0.97]	1.36 [0.63-2.96]
Females	2.01 [0.23-17.7]	1.05 [0.65-1.70]	1.27 [0.68-2.37]

[a] Adjusted for gestational age, birth length, birth weight, age at start, height at start, parental educational level, family income and sex

Figure S1: Kaplan-Meier curves of overall neoplastic events, separated by sex

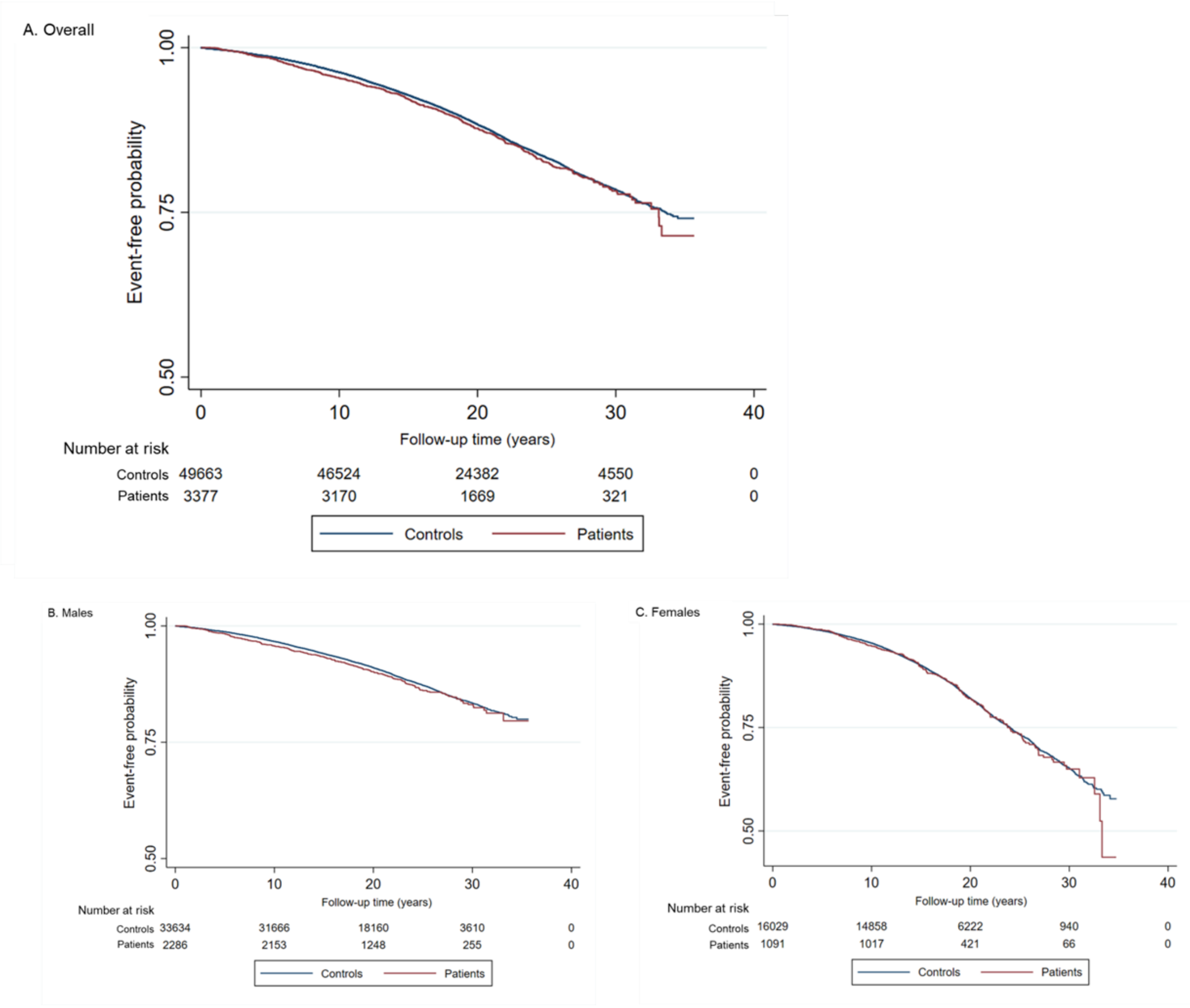

Figure S2: Kaplan-Meier curves of malignant neoplastic events, separated by sex

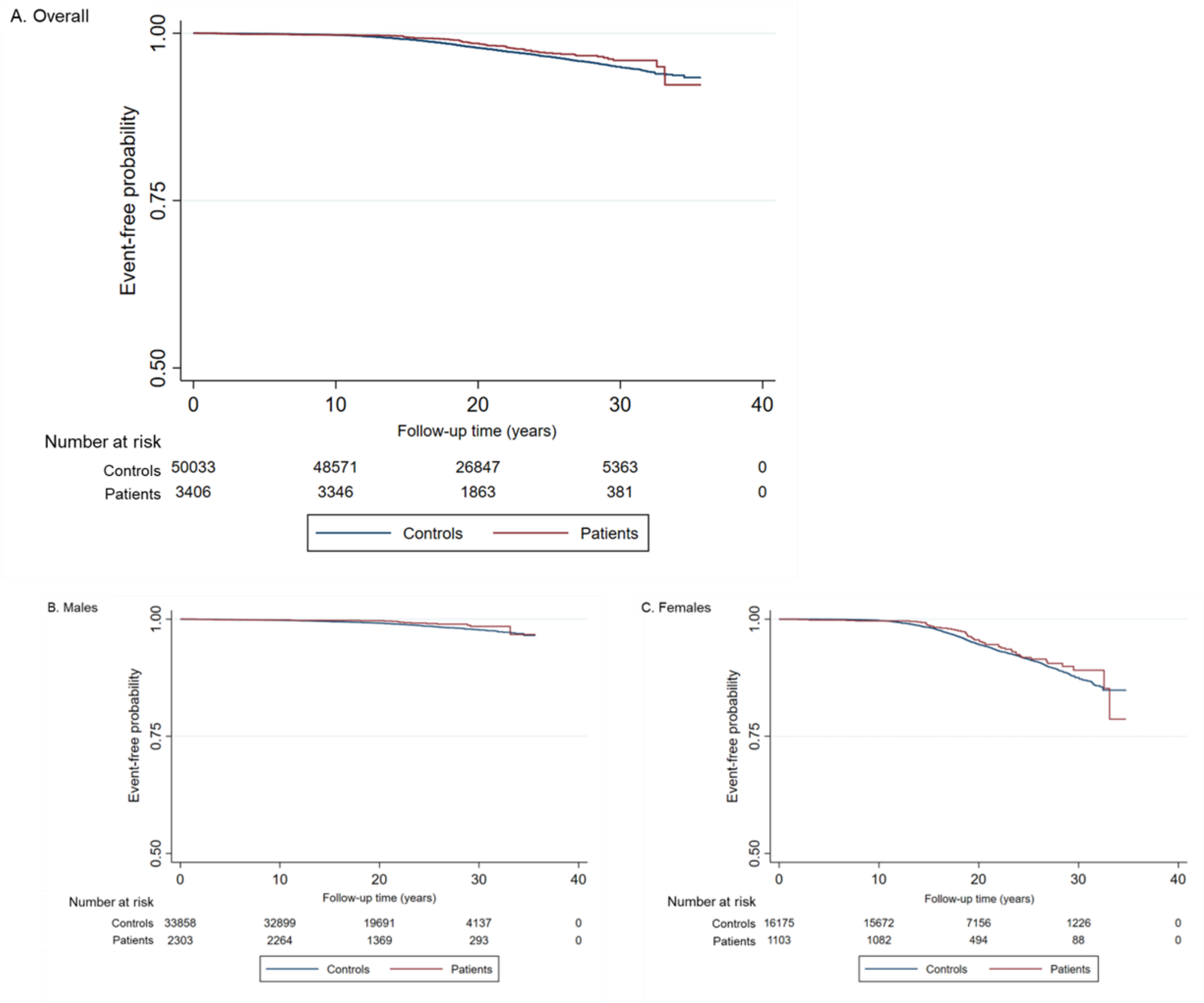

Supplement: Supplementary file 1 [file DataSheet_1.pdf]
